# 

**Published:** 1857-10

**Authors:** 


### CONTENTS.

ORIGINAL COMMUNICATIONS— PAGE.

Report of Surgical Cases, treated at St. James' Hospital, Chicago, during the months of July and August. By Dr. Geo. K. Amerman, one of the attending Surgeons ........ 439
Valerianate of Ammonia in the Treatment of Neuralgia. By W. Hay, M. D., Chicago ........ 444
Ligaturing one of the common Corotid Arteries, for the cure of Epilepsy. By C. Angell, of Pittsburgh, Indiana ........ 446
A Case of Paralysis. By Charles Brackett, M. D., of Rochester, N. Y. 448
A Communication. By S. Ramsey, Reedsburg, Wis. ........ 449

PROCEEDINGS OF MEDICAL SOCIETIES—

Meeting of the Cook County Medical Society, ........ 451

SELECTIONS—

Dr. Edward Brown-Sequard's Experimental and Clinical Researches—Epilepsy—(continued), ........ 452

BOOK NOTICES—

The Physiological Anatomy and Physiology of Man. By Robert Bently Todd, M. D., F. R. S., etc., etc., and William Bowman, F. R. S., etc., etc. Complete in one volume, with two hundred and ninety-eight illustrations. Blanchard and Lea, Philadelphia. ........ 462
Transactions of the South Carolina Medical Association, at the Extra Meeting in Sumpter, and at the Annual Meeting in Charleston ........ 468

EDITORIAL—

Biographical Sketch of Dr. Marshall Hall ........ 470
A Word to our Subscribers ........ 478
Rush Medical College ........ 478
Medical Education ........ 479
Clinical Instruction ........ 479
Epidemic Cholera ........ 480
Hydrophobia ........ 481

ADVERTISEMENTS—

To the Medical Profession, ........ 483
Medical College of Ohio, ........ 485
Starling Medical College ........ 486

